# Baihu Jia Guizhi Decoction Improves Rheumatoid Arthritis Inflammation by Regulating Succinate/SUCNR1 Metabolic Signaling Pathway

**DOI:** 10.1155/2019/3258572

**Published:** 2019-12-26

**Authors:** Huan Chen, Ting Pan, Panwang Liu, Ping Wang, Shijun Xu

**Affiliations:** ^1^Institute of Material Medical Integration and Transformation for Brain Disorders, Chengdu University of Traditional Chinese Medicine, Chengdu 611137, China; ^2^School of Pharmacy, Chengdu University of Traditional Chinese Medicine, Chengdu 611137, China

## Abstract

Rheumatoid arthritis (RA) is an autoimmune disease characterized by synovitis. Succinate is an inflammatory metabolic signal that exacerbates RA synovitis by activating succinate receptor 1 (SUCNR1) to amplify the release of IL-1*β*. Thus, inhibition of succinate activation of SUCRN1 could be an effective method to inhibit the inflammation of RA. Baihu Jia Guizhi decoction (BHGZ), which is composed of *Gypsum Fibrosum*, *Anemarrhena asphodeloides* Bge., *Cinnamomum cassia* Presl., *Glycyrrhiza uralensis* Fisch., and *Oryza sativa* L., is a Traditional Chinese Medicine (TCM) prescription used to treat RA in clinic. In addition, TCM believes that damp and heat environment is one of the causes of RA. In this study, we tested the role of damp and heat environments in exacerbating RA inflammation and the anti-inflammatory effect of BHGZ, based on succinate/SUCNR1/IL-1*β* pathway in the adjuvant arthritis (AA) model with damp and heat environment (AA + DHE). Results showed that paw swelling and synovial pathology were significantly increased in AA rats, and these results were aggravated by stimulation in damp and heat environment. BHGZ improved AA + DHE rats' paw swelling, synovial hyperplasia, and inflammatory cell infiltration and reduced IL-1*β*. In addition, AA rats significantly increased the expression of SUCNR1, and the stimulation of damp and heat environment not only increased the expression of SUCNR1 but also promoted the accumulation of succinate. BHGZ simultaneously reduced the concentration of succinate and the expression of SUCNR1. Finally, SDH activity was decreased in AA rats and AA + DHE rats, while BHGZ increased SDH activity and then reduced succinate concentration. Therefore, we prove that damp and heat environment deteriorated the inflammation of RA which is the activation of succinate/SUCNR1 pathway, while BHGZ regulates SDH activity to reduce the accumulation of succinate and inhibit the activation of SUCNR1 that is the underlying mechanism of its treatment of RA.

## 1. Introduction

Rheumatoid arthritis (RA) is a systemic autoimmune inflammatory disease characterized by synovitis, peripheral joint inflammation, and progressive destruction of cartilage and bone [1]. Nearly 1% people in the world suffer from RA [2]. Studies on the pathogenesis of RA indicate that synovial compartment is the key site for the pathogenesis of RA. With the help of chemokines, leukocytes are migrated to the synovium, causing synovial inflammatory infiltration and proliferation of fibroblast-like synoviocytes (FLSs) which induced synovial hyperplasia [3]. In addition, immune cells within the synovial membrane release many inflammatory mediators, such as interleukin- (IL-)1, IL-17, and tumor necrosis factor *α* (TNF-*α*), that produce an inflammatory cascade. Finally, the synovial fluid is filled with inflammatory mediators leading to cartilage and bone damage. Clinical studies have found that a large amount of succinate, which is a metabolic intermediate of the Krebs cycle, appeared in the joints of patients with RA [4]. In recent years, experimental studies have shown that excessive accumulation of succinate can aggravate joint inflammation and vascular hyperplasia [5]. Plenty of evidences suggest that abnormal energy metabolism can contribute to the aggravation of RA inflammation and cartilage damage [5–7]. This abnormal energy metabolism includes hyperactive glycolysis and abnormal Krebs cycle in synoviocytes and macrophages. In particular, this abnormal energy metabolism leads to massive accumulation of succinate and lactic acid in synovial fluid of RA patients [8, 9].

Succinate exerts a proinflammatory effect, both extracellularly and intracellularly. Intracellular succinate promotes transcription of inflammatory factors by stabilizing hypoxia-inducible factor 1*α* (HIF-1*α*) and extracellular succinate activation succinate receptor 1 (SUCNR1), which leads to calcium activation and protein kinase C (PKC) activation and eventually releasing nitric oxide (NO) and prostaglandin E2 (PGE2).

In macrophages, the release of IL-1*β* is thought to be dependent on SUCNR1 activation [10]. Given that the combination of succinate and SUCNR1 induce the release of IL-1*β*, resulting in a dramatic amplification of inflammatory response of RA [10], scientists at the Novartis Institute for Biomedical Research revealed that succinate-binding receptor SUCNR1 can be used as a potential therapeutic target [10]. Meanwhile, SUCNR1 antagonists have been studied in many fields, such as Advinus Therapeutics which is working on SUCNR1 antagonists for eye and liver diseases.

Environmental factors are considered as important causes of RA [11]. Clinical studies have shown that RA patients living in inappropriate environments, such as high temperature and high humidity, will aggravate pain of joints [12]. Our previous studies have shown that high temperature (37 ± 1°C) and high humidity (relative humidity of 70–80%) environment aggravate synovial joint lesions in rats with adjuvant arthritis (AA) (relevant content published in another article). In Traditional Chinese Medicine, Baihu Jia Guizhi decoction (BHGZ), which has been recorded in *Jin Gui Yao Lue* more than 1800 years, is the first choice for treating RA caused by damp and heat environment. Previous pharmacological research points out that BHGZ can significantly improve the joint inflammation response in RA rats [13, 14]. The 5 herbs of BHGZ include *Gypsum Fibrosum*, *Anemarrhena asphodeloides* Bge., *Cinnamomum cassia* Presl., *Glycyrrhiza uralensis* Fisch., and *Oryza sativa* L. It has been proved that the main herbs and their compounds in BHGZ can inhibit the inflammatory response of various diseases from different mechanisms [15–20]. However, the underlying mechanism behind the inhibition of inflammation by BHGZ is still unclear. In this study, we explored the potential mechanism of BHGZ in the treatment of RA by regulating the abnormal accumulation of succinate and its mediated downstream signal pathway.

## 2. Materials and Methods

### 2.1. Reagents

Mangiferin, liquiritin, cinnamic acid, cinnamaldehyde, timosaponin BII, and monoammonium glycyrrhizinate were brought from Chenguang Biotechnology Co., Ltd. (Xian, China). Complete Freund's Adjuvant (CFA) was purchased from Sigma (Sigma Chemical Co., USA). The ELISA kit of IL-1*β* (2301B70543) was brought from Multi Sciences (Lianke) Biotech Co., Ltd. (Hangzhou, China). The Succinate dehydrogenase (SDH) kit (A022) was purchased from Nanjing Jiancheng Bioengineering Institute (Nanjing, China). The anti-SUCNR1 antibody (ab140795) was obtained from Abcam (Cambridge, MA, USA). The goat anti-rabbit horseradish peroxidase-linked antibody kit (SP9001) was brought from zhongshan Jinqiao Biotechnology Co., Ltd. (Beijing, China). The standard of succinate, pyruvic acid, and fumaric acid was obtained from Chengdu Herb Purify Co., Ltd. (Sichuang, China).

### 2.2. Preparation of Baihu Jia Guizhi Decoction

All herbs needed for BHGZ were purchased from Chinese natural herbal special market at Sichuan Chengdu, China. BHGZ contain five components: *Gypsum Fibrosum*, *Anemarrhena asphodeloides* Bge., *Cinnamomum cassia* Presl., *Glycyrrhiza uralensis* Fisch., and *Oryza sativa* L. in 12 : 3 : 2 : 1 : 6 ratio. All herbs were identified by Professor Yuntong Ma, an expert in pharmacognosy (Chengdu University of Traditional Chinese Medicine). The preparation method of BHGZ extractions followed the previous study [14]. The specific operation was described below: *Gypsum Fibrosum* was decocted to boiling for 30 min, and then three other crude drugs were added, which had been soaked in warm water (1 : 8, w/v) for 30 min and *Oryza sativa* L. and decocted to boiling for 30 min. The decoction was filtered through a four-layer gauze. Later, the drug residue was boiled once again in water (1 : 5, w/v) for 30 min. The filtrates were merged twice and concentrated under reduced pressure to a concentration of 2 g/ml. Finally, the filtrate was stored at 4°C. The quality of BHGZ was measured by high-performance liquid chromatography (HPLC). The detection condition was as follows: column: Ultimate AQ-C18 Column (4.6 mm × 25 *μ*m, 5 *μ*m); mobile phase: A for 0.01% formic acid water and B for acetonitrile; gradient elution: (0–15 min, 5% B; 15–17 min, 5–10% B; 17–22 min, 10–17% B; 22–24 min, 17–19% B; 24–33 min, 19–20% B; 33–45 min, 20%–35% B; 45–55 min, 35%–42% B; 55–58 min, 42%–42% B; 55–58 min, 42%–42% B; and 58–65 min, 42%–5% B); flow rate: 1.0 ml/min; detection wavelength: 255 nm; injection volume: 10 *μ*l; and column temperature: 24°C. In addition, Ca^2+^ concentration in 1 mg/ml of BHGZ was measured by an electrolyte analyzer (Caretium Medical Instruments Co., Ltd., Shenzhen, China).

### 2.3. Animals

Male Sprague-Dawley rats (*n* = 56), 6 weeks of age, were obtained from the Institute of Laboratory Animals of Sichuan Academy of Medical Sciences (no. 0010716, Chengdu, China). All rats were housed in room temperature 22–26°C and relative humidity 55 ± 5% for 3 days before experiment. All experiments were fulfilled in accordance with the guidelines of the Care and Use of Laboratory Animals published by the National Institute of Health (NIH publication no. 85-23, revised 1996), and all experimental operations were approved by the Institutional Ethics Committee of Institute of Material Medical Integration and Transformation for Brain Disorder, Chengdu University of Traditional Chinese Medicine (no. IBD2017017).

### 2.4. Induction of Animal Model and Treatment

Before the injection of CFA, all animals were randomly divided into seven groups, 8 animals for each group. The animal model of adjuvant arthritis (AA) was induced by intradermal injection of 0.1 ml CFA into the right hind metatarsal footpad, as described previously [21]. The damp and heat environment model of RA was established on the AA model, which was put into intelligent artificial climate incubator at a temperature of 37 ± 1°C and a relative humidity of 70% to 80%, 3 h a day for 6 weeks, which round from 9 am to 12 pm [14]. In this paper, we abbreviated the damp and heat environment model of RA to AA + DHE. After the injection of CFA two weeks, AA + DHE rats were orally administered by BHGZ (28 g/kg and 14 g/kg and 7 g/kg) once a day, for 4 weeks. The dose of BHGZ was calculated based on the daily dosage of adults recommended by the TCM prescription and clinical manual and the surface area ratio of human and rats. Dexamethasone (DXMS 0.08 mg/kg), acted as positive drugs, was given to the rats of AA + DHE by orally administered, once a day. The dose of DXMS was calculated according to the adult daily maintenance dose. The normal group, AA group, and AA + DHE group were orally administered with distilled water simultaneously.

### 2.5. Evaluation of Arthritis

Plethysmometer paw volume (Taimeng Technology, Chengdu, China) was used to evaluate the paw swelling of rats. On days 0, 7, 14, 21, 28, 35, and 42 after immunization, the right hind paw of rat was detected. Each paw was measured twice, and the average data were noted as the final record. Right paw swelling perimeter was measured by a soft tape on day 0, 7, 14, 21, 28, 35, 42. The change of paw swelling volume (△paw swelling volume) and paw swelling perimeter (△paw swelling perimeter) was calculated by *V*_*t*_ − *V*_0_ (ml) and *P*_*t*_ − *P*_0_ (cm), respectively. *V*_0_ and *P*_0_ are mean paw swelling volume and perimeter before the injection of CFA, and *V*_*t*_ and *P*_*t*_ are paw swelling volume and perimeter at *t* day after injection.

### 2.6. Histological Analysis

Ankle samples fixed in 4% paraformaldehyde were washed by water, dehydrated with ethanol, and embedded in paraffin. Then, the paraffin sections which had only 5 *μ*m thickness were deparaffinized with xylene and ethanol and stained with hematoxylin and eosin.

### 2.7. Rats IL-1*β* Assay in Serum

Serum samples were collected from the whole blood through centrifuged 3000 rpm for 10 min and stored at −80°C immediately. According to the manufacturer's instruction of IL-1*β* ELISA kit to detect the level of IL-1*β* in serum.

### 2.8. Measurement of Succinate, Pyruvate, and Fumarate in Synovial Tissue

The synovial tissue was ground at low temperature with liquid nitrogen, and 10 times volume of normal saline was added to prepare synovial homogenate. Then, 250 *μ*l perchloric acid was added (0.5 mol/L) into 250 *μ*l homogenate, vortexed for 1 min, and allowed to stand on ice for 10 min. The homogenate was centrifuged at 4°C for 20 min at a speed of 13000*g*, the supernatant was collected, centrifuged again for 10 min, and 10 *μ*l of the supernatant was taken for HPLC. The HPLC mobile phase was a phosphate buffer (pH 2.5) at a flow rate of 1 ml/min and a detection wavelength of 210 nm [22]. Pure water is used to dissolve succinate, pyruvate, and fumarate standard products.

### 2.9. Rats SDH Activity Assay in Synovial Tissue

The method of preparing synovial homogenate is described above. The activity of SDH in synovial tissue was measured by SDH commercial kit according to the manufacturer's instructions.

### 2.10. Immunohistochemistry

The paraffin-embedded tissues were cut into 4 *μ*m slices, the anti-SUCNR1 antibody was incubated according to the manufacturer's specifications, and the slices were counterstained with hematoxylin. Finally, the microscope was used to take pictures, and the Image-Pro plus software was used to measure the average optical density.

### 2.11. Statistical Analysis

Data of this study were expressed as mean ± standard deviation (SD). One-way ANOVA and Mann–Whitney *U* test were used to analyze statistical differences between groups. A value of *P* < 0.05 was considered statistically significant.

## 3. Results

### 3.1. The Concentration of Representative Components in BHGZ

**Figure 1:**
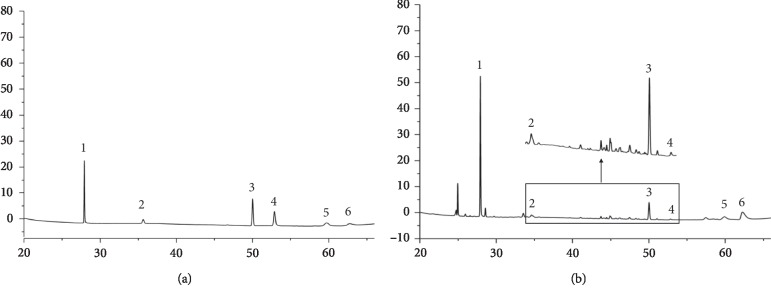
Typical chromatogram of BHGZ. (a) The peak of six standards: 1, mangiferin (0.036 mM); 2, liquiritin (0.015 mM); 3, cinnamic acid (0.018 mM); 4, cinnamaldehyde (0.003 mM); 5, timosaponin BII (0.068 mM); 6, monoammonium glycyrrhizinate (0.011 mM). (b) HPLC profile of BHGZ.

The representative chemical components in BHGZ were detected by HPLC and an electrolyte analyzer. The peak retention time and concentration of each chemical component are shown in Figure 1 and Table 1.

### 3.2. Effect of BHGZ on the Change of Body Weight

**Figure 2:**
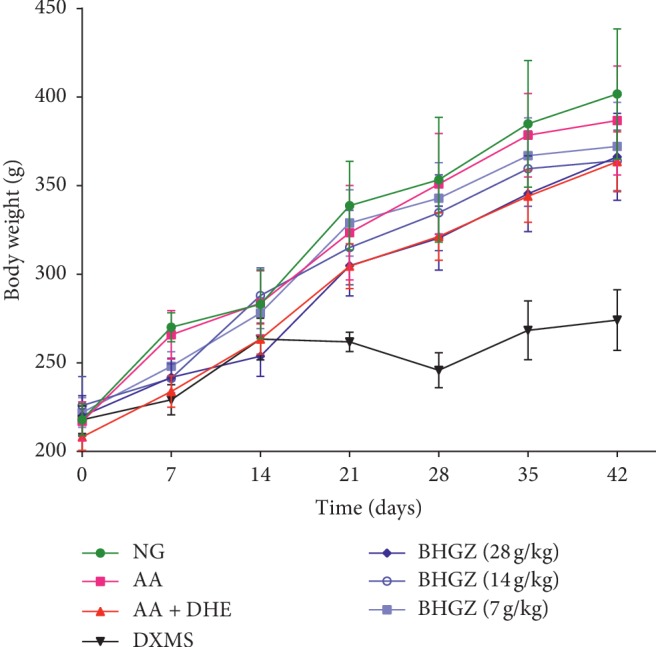
Effect of BHGZ on the change of body weight. Body weight of each group was measured on a weekly basis. (*n* = 8). BHGZ, Baihu Jia Guizhi decoction; AA, adjuvant arthritis; AA + DHE, AA with damp and heat environment.

As shown in Figure 2, except for the DXMS group, the weight of each group showed a gradual increase. No significant differences were observed in body weight among NG, AA, AA + DHE, and BHGZ administration groups. However, the weight gain of the rats after the damp and heat environment stimulated was lower than that of the NG and AA group.

### 3.3. Effects of BHGZ on the Change of Paw Swelling Volume and Perimeter

**Figure 3:**
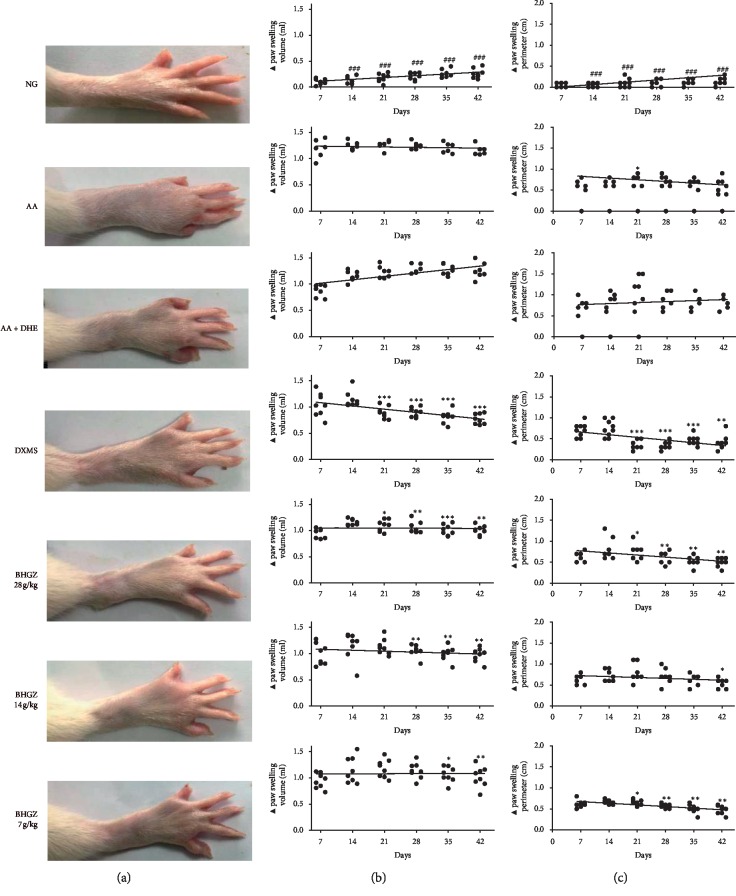
Effects of BHGZ on the change of paw swelling volume and perimeter. Image analysis: (a) the paw swelling of rats; (b) the change of paw swelling (▲paw swelling volume); (c) the change of paw swelling perimeter (▲paw swelling perimeter) (*n* = 8). ^###^*P* < 0.001 illustrated the extraordinary difference compared with the AA group; ^*∗*^*P* < 0.05, ^*∗∗*^*P* < 0.01 and ^*∗∗∗*^*P* < 0.001 represent statistically significant differences compared with the AA + DHE group. BHGZ, Baihu Jia Guizhi decoction; AA, adjuvant arthritis; AA + DHE, AA with damp and heat environment.

The therapeutic effect of BHGZ on AA + DHE rats was studied by evaluating the changes of paw swelling volume and perimeter over time. Macroscopic evaluation of the paw swelling of AA group and AA + DHE group rats showed that all these rats injected with CFA revealed obvious paw swelling during the whole experiment, but the AA + DHE group rats stimulated by dump and heat environment revealed more erythema than the AA rats, and some rats appeared with joint sclerosis (Figure 3(a)). The results of the change of paw swelling volume and perimeter are shown in Figures 3(b) and 3(c). In 42-day experiment, the changes of paw swelling volume and perimeter of AA + DHE group rats showed an increasing trend with time, while the changes of paw swelling volume of AA rats remained stable, and the changes of paw swelling perimeter showed a decreasing trend. This indicated that the foot swelling of AA + DHE group rats was more serious than that of AA rats. However, after one week of BHGZ treatment, paw swelling was significantly suppressed. But, the effects of three doses of BHGZ on paw swelling and paw perimeter were similar, with no significant dose-dependence observed.

### 3.4. Effects of BHGZ on Synovial Histopathological Changes

**Figure 4:**
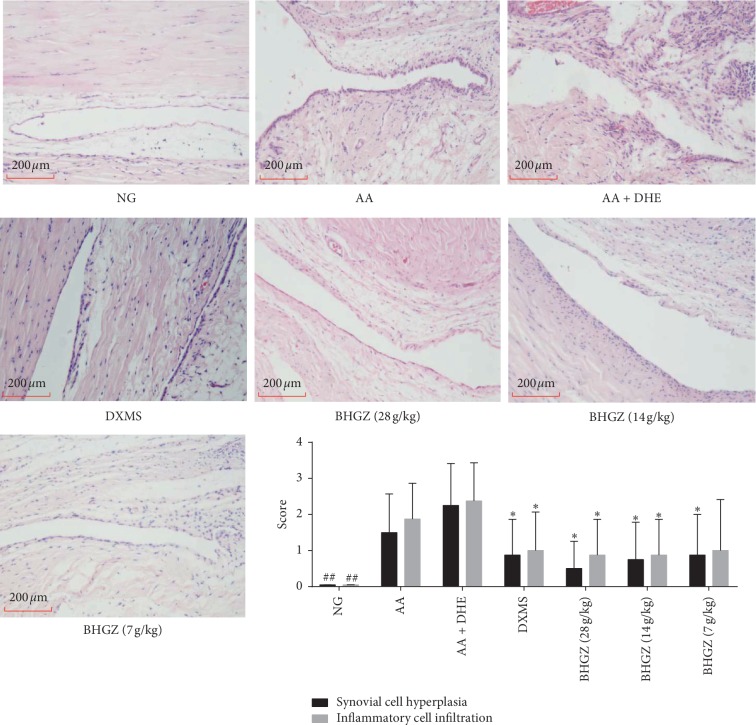
Effects of BHGZ on synovial histopathological changes. On the 42^nd^ day of the experiment, the right ankle joint of the rat was cut and fixed with paraformaldehyde for pathological examination of HE. The pathological statistical scores included synovial hyperplasia and inflammatory cell infiltration. Data are mean ± S.D. (*n* = 8). ^##^*P* < 0.01 illustrated the extraordinary difference compared with the AA group; ^*∗*^*P* < 0.05 and ^*∗∗*^*P* < 0.01 represent statistically significant differences compared with the AA + DHE group. BHGZ, Baihu Jia Guizhi decoction; AA, adjuvant arthritis; AA + DHE, AA with damp and heat environment.

Synovial histopathological changes are the most important indicators to determine the success of AA model and whether the drug has any effect. Pathological results (Figure 4) indicated that the synovial tissues in the normal group did not possess synovial hyperplasia, fibrosis, cell swelling, and inflammatory cell infiltration. Nevertheless, synovial hyperplasia and inflammatory cell infiltration were observed in the AA group. The synovial hyperplasia and inflammatory cell infiltration of the AA + DHE group were mildly serious than the AA group. However, synovial hyperplasia and inflammatory cell infiltration were decreased about 50% by BHGZ (28, 14, 7 g/kg) compared with the AA + DHE group rats.

### 3.5. Effects of BHGZ on the Content of IL-1*β* in Serum

**Figure 5:**
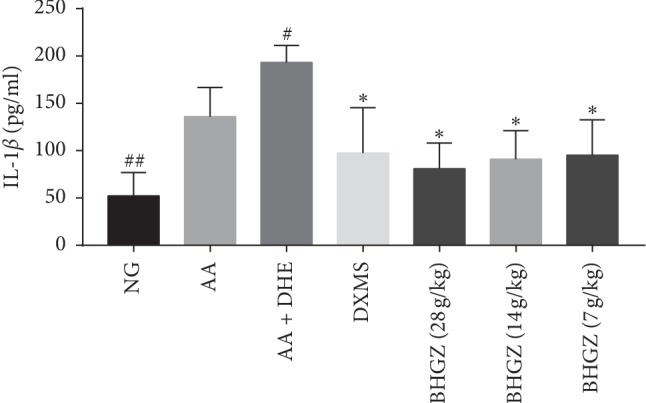
Effects of BHGZ on the content of IL-1*β* in serum. Data are mean ± S.D. (*n* = 8). ^##^*P* < 0.01 illustrated the extraordinary difference compared with the AA group; ^*∗*^*P* < 0.05 represents statistically significant differences compared with the AA + DHE group. BHGZ, Baihu Jia Guizhi decoction; AA, adjuvant arthritis; AA + DHE, AA with damp and heat environment.

As shown in Figure 5, compared with the normal group, the concentration of IL-1*β* was significantly augment in AA and AA + DHE groups. Moreover, IL-1*β* of *d* AA + DHE group was conspicuous aggrandized compared with the AA group. However, compared with the AA + DHE group, BHGZ (28, 14, 7 g/kg) remarkably reduced the concentration of IL-1*β*.

### 3.6. Effects of BHGZ on Succinate/SUCNR1 in Synovial Tissue

**Figure 6:**
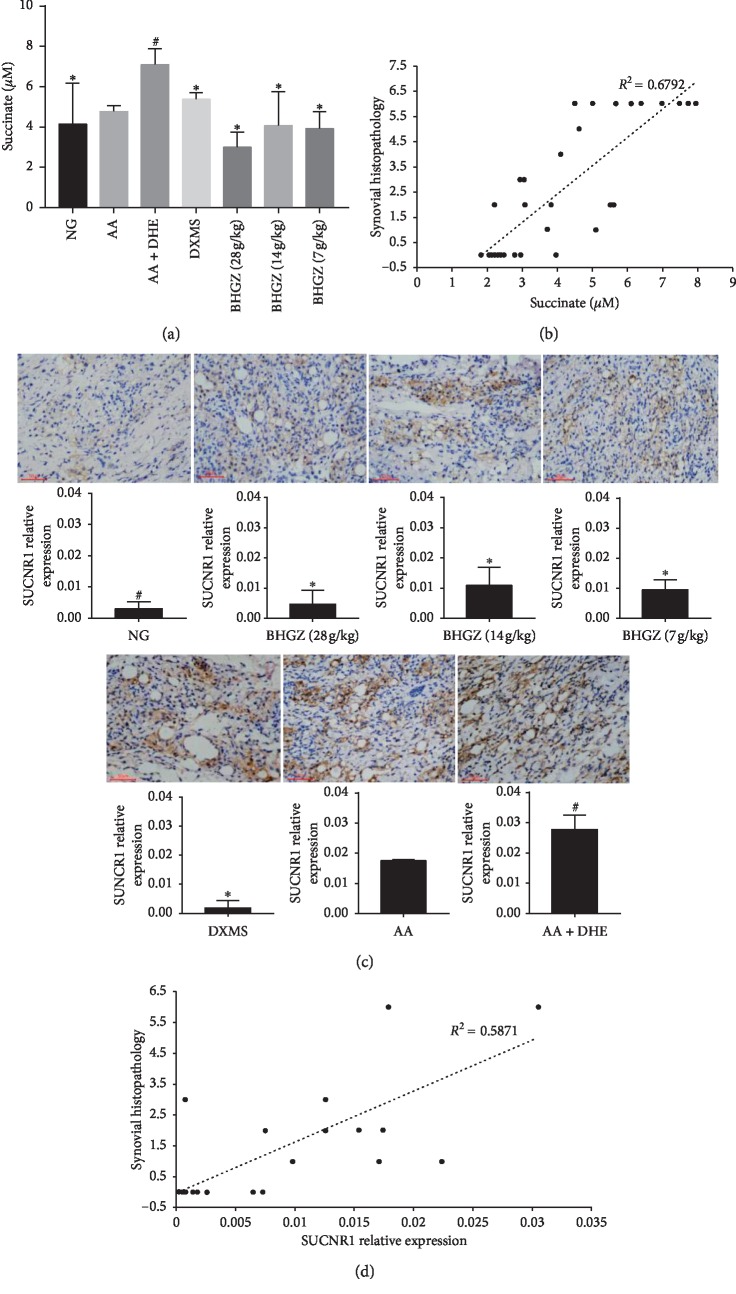
Effects of BHGZ on succinate and the expression of SUCNR1. On the 42^nd^ day of the experiment, the right ankle joint of the rats was cut and fixed with paraformaldehyde for immunohistochemical detection. (a) The content of succinate in synovial tissue (*n* = 5). (b) Correlation analysis of succinate and pathology. (c) Representative images of SUCNR1 immunohistochemistry in each group were exhibited (*n* = 3). (d) Correlation analysis of the expression of SUCNR1 and pathology. Data are mean ± S.D. ^##^*P* < 0.01 illustrated the extraordinary difference compared with the AA group; ^*∗*^*P* < 0.05 and ^*∗∗*^*P* < 0.01 represent statistically significant differences compared with the AA + DHE group. BHGZ, Baihu Jia Guizhi decoction; AA, adjuvant arthritis; AA + DHE, AA with damp and heat environment.

We examined the concentration of succinate and the expression of SUCNR1 in synovial tissue. The chromatogram of succinate in synovial tissue was shown in Supplementary [Supplementary-material supplementary-material-1]. HPLC analysis results showed that succinate did not increase significantly after CFA injection, but increased significantly after damp and heat conditions (Figure 6(a)). However, succinate was significantly reduced after BHGZ treatment, in which the effect of high dose was 2.37 times lower than that of the AA + DHE group (Figure 6(a)). In addition, to study the relationship between succinate and the severity of the pathology in the synovial tissue, we conducted a correlation analysis between the two. Results are shown in (Figure 6(b)); the correlation between succinate and pathology, including inflammatory cell infiltration and synovial cell proliferation, was as high as 0.6792. This suggests that succinate is closely related to synovial pathological changes.

Subsequently, the expression of SUCNR1 in synovial tissue was detected by immunohistochemistry. Results are shown in Figure 6(c); after CFA immunization, the expression of SUCNR1 increased by 5.8 times, while the expression of SUCNR1 was further promoted after damp and heat environment stimulation, which was nearly 28 times of that in normal group. However, after treatment with BHGZ, the expression of SUCNR1 decreased significantly. The multiple of reducing SUCNR1 expression in each drug group was 18 times of BHGZ (28 g/kg), 8 times of BHGZ (14 g/kg), and 9 times of BHGZ (7 g/kg). Moreover, we also analyzed the relationship between SUCNR1 expression and pathological changes in synovial tissue, and results showed that the correlation between SUCNR1 expression and pathological severity was 0.5871 (Figure 6(d)).

### 3.7. Effects of BHGZ on Pyruvate, Fumarate, and SDH in Synovial Tissue

**Figure 7:**
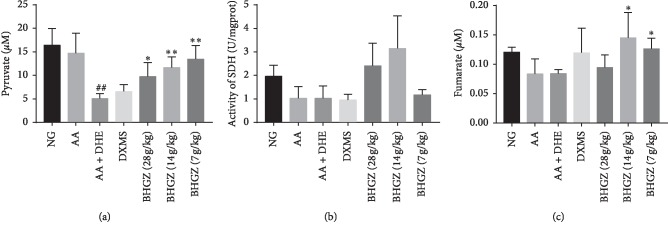
Effects of BHGZ on pyruvate, fumarate, and the activity of SDH. Data are mean ± S.D. (a) The content of pyruvate in synovial tissue (*n* = 5). (b) The activity of SDH in synovial tissue (*n* = 3). (c) The content of fumarate in synovial tissue (*n* = 5). ^#^*P* < 0.05 and ^##^*P* < 0.01 illustrated the extraordinary difference compared with the AA group; ^*∗*^*P* < 0.05 and ^*∗∗*^*P* < 0.01 represent statistically significant differences compared with the AA + DHE group. BHGZ, Baihu Jia Guizhi decoction; AA, adjuvant arthritis; AA + DHE, AA with damp and heat environment.

In this part of the experiment, we explored the reason for the accumulation of succinate in synovium. The chromatograms of pyruvate and fumarate in synovial tissue were shown in Supplementary [Supplementary-material supplementary-material-1]. Firstly, pyruvate is the product of glycolysis, which can enter the TCA cycle oxidation decomposition and affects the content of metabolites in the TCA cycle. HPLC test results (Figure 7(a)) showed that the pyruvate did not change significantly after CFA immunization, but the pyruvate decreased by 2.4 times in AA + DHE group rats, while after BHGZ administration, pyruvate increased significantly and was negatively correlated with the dose. In addition, SDH is the enzyme that hydrolyzes succinate into fumarate in TCA cycle, so its enzymatic activity is closely related to the succinate concentration. As shown in Figure 7(b), there was no statistically different SDH enzyme activity in AA group and AA + DHE group rats, compared with normal group, but a downward trend could be seen. After BHGZ administration, the activity of SDH enzyme increased. It was suggested that the decrease of succinate in synovial tissue by BHGZ was related to the increase of SDH enzyme activity. Finally, we also tested the concentration of fumarate in the hydrolytic products of succinate. The results showed that fumarate was significantly reduced in the AA group and AA + DHE group, while BHGZ significantly increased fumarate (Figure 7(c)). This may be related to the increased SDH enzyme activity of BHGZ.

## 4. Discussion

RA researchers have been found that the abnormally increased succinate, which is a metabolic intermediate of the tricarboxylic acid cycle, is one of pivotal reasons for the aggravation of RA [23]. Studies have confirmed that succinate can aggravate RA joint inflammatory response by promoting the release of inflammatory mediator IL-1*β* and increasing synovial angiogenesis [5, 10]. Inflammation is a major feature of RA, while synovial angiogenesis promotes inflammatory cell migration and angioplasty, ultimately damaging cartilage and bone [24, 25]. In the present study, through correlation analysis, we confirmed that the accumulation of succinate promoted synovial injury of RA. SDH catalyzes the hydrolysis of succinate into fumarate to avoid excessive accumulation of succinate. In this study, we found that although there was no statistical difference of SDH between each group, the activity of SDH shown a clear downward trend in AA and AA + DHE model groups.

In inflammatory response, succinate acts as a ligand to activate the G protein-coupled receptor (GPCR) GPR91, since renamed SUCNR1. A study indicated that succinate acts as a chemokine which induce dendritic cell (DC) migrating to lymphoid nodes [26]. While after lacking SUCNR1, the migration of DC decreased. Succinate can also enhance the antigen presentation capacity of DC to promote the activation of T cells and produce TNF-*α* and interferon-*γ* (IFN-*γ*) [26]. This process was eliminated in SUCNR1 deficient DC. These studies confirmed that SUCNR1 was essential in immune cell inflammation and the increase of antigen presentation caused by succinate [26]. In addition, a recently study shown that succinate stimulates the bone-marrow-derived macrophage (BMDM) to release the IL-1*β*, which relies on the activation of SUCNR1 [10]. In this study, we found that the expression of SUCNR1 and IL-1*β* significantly increased after CFA immunization. The increase of IL-1*β* release may be related to the activation of downstream phospholipase C (PLC) by SUCNR1. The activation of the PLC leads to calcium and PKC activation, finally activating the nuclear factor *κ*B (NF-*κ*B) pathway and MAPK cascade [27].

Recent research suggests that environment has been widely recognized as a predisposing factor of RA. As early as 2500 years ago, a book named *Su Wen* in Traditional Chinese Medicine described environmental factors as an important catalyst for RA [28]. The mechanism of most environmental factors on RA is still unclear. An interesting hypothesis is that environmental factors may influence RA immune response by altering epigenetic modifications [29]. Previous research indicated that AA rats could change DNA methylation and metabolomics under the stimulation of damp and heat environment (this part of the experiment by our team is being prepared in another article). In this article, AA rats were stimulated by damp and heat environment to explore the influence of environment on the progression of RA disease. Finally, our research shows that damp and heat environment significantly affected the metabolic intermediates of tricarboxylic acid cycle in synovial tissue, including increasing succinate, reducing pyruvate, and fumarate. These results indicate that damp and heat environment promoted RA which might be related to the influence of energy metabolism.

Previous research has focus on anti-inflammation and immune regulation to state the anti-RA mechanism of BHGZ. In this paper, we investigated the upstream mechanism of BHGZ-inhibiting inflammatory response. We found that BHGZ improved paw swelling and synovial pathology and was associated with inhibition of the activation of the succinate/SUCNR1 signaling in synovial tissues. Furthermore, BHGZ reduced the accumulation of succinate by increasing SDH enzyme activity. Our results also showed that BHGZ regulated the contents of succinate, pyruvate, and fumarate, all of which are important metabolic intermediates in the process of energy production. Therefore, we concluded that BHGZ may regulate energy metabolism in RA synovial membrane. Literature studies have shown that all the constituent herbs in BHGZ can regulate energy metabolism. The composition of BHGZ is to add *Cinnamomum cassia* Presl., to Baihu Tang which includes *Gypsum Fibrosum*, *Anemarrhena asphodeloides* Bge., *Glycyrrhiza uralensis* Fisch., and Oryza sativa L. The study suggested that Baihu Tang enhanced glucose uptake by activating peroxisome proliferator-activated receptor gamma (PPAR*γ*) in 3T3-L1 adipocytes [30]. In addition, Baihu Tang increased the activation of catalytic and oxidoreductase for metabolic processes at the intracellular part [31]. Studies on *Gypsum Fibrosum* have found that gypsum enhanced the activation of PKC, which reduces the activation of sodium-dicarboxylate cotransporters 3 (NaDC-3) to downregulate the transport of succinate [32, 33]. Studies have found that *Anemarrhena asphodeloides* Bge., can improve glucose and lipid metabolism by regulating the activation of AMP-activated protein kinase in diabetic mice [34]. Cinnamaldehyde and cinnamomol are main active substances in *Cinnamomum cassia* Presl., and can significantly regulate glucose and lipid metabolism [35–38]. Studies shown that the active ingredient of *Glycyrrhiza uralensis* Fisch. has an effect of glucose metabolism, such as isoliquritigenin inhibiting glycolysis in mouse melanoma cells, amorfrutins regulateing peroxisome proliferator-activated receptor (PPAR) activity to exert antidiabetes effect, and glabridin activating AMP to induce glucose uptake [39–41]. Therefore, based on the above studies, we believe that BHGZ may play a role in regulating energy metabolism. This also provides a new research direction for our follow-up studies on BHGZ improving RA.

## 5. Conclusion

**Figure 8:**
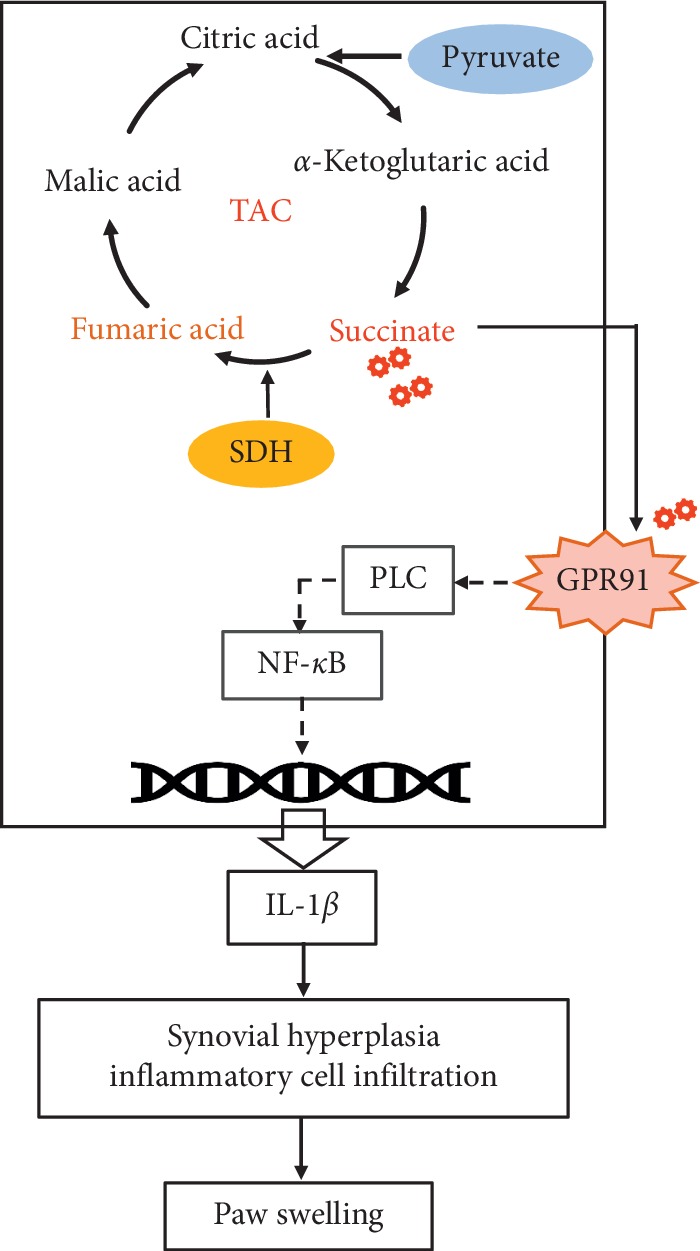
BHGZ reducing the accumulation of succinate by increasing the activity of SDH and inhibiting the expression of SUCNR1 to downregulate IL-1*β* and finally improving synovial pathology.

BHGZ, composed of *Gypsum Fibrosum*, *Anemarrhena asphodeloides* Bge., *Cinnamomum cassia* Presl., *Glycyrrhiza uralensis* Fisch., and *Oryza sativa* L., is a classic TCM prescription used to treat RA in clinic. In this study, we used BHGZ to treat adjuvant-induced arthritis rats, which is a common animal model of RA. BHGZ can significantly improve paw swelling and synovial pathology in this animal model. Its potential therapeutic mechanism may be related to reduce succinate by increasing SDH activity and inhibiting SUCNR1 protein expression and to downregulate inflammatory factors IL-1*β*. BHGZ treatment mechanism diagram is shown in Figure 8.

## Figures and Tables

**Table 1:** The concentration of representative components in BHZG.

Standard substance	Concentration (*μ*M)
Mangiferin	859
Liquiritin	1.4
Cinnamic acid	11.6
Cinnamaldehyde	0.15
Timosaponin BII	2726
Monoammonium glycyrrhizinate	33.8
Ion Ca^2+^	1770
Total Ca^2+^	3460

## Data Availability

The data used to support the findings of this study are available from the corresponding author upon request.

## Abbreviations

AA: Adjuvant arthritis
AA + DHE: AA rats with damp and heat environment stimulating
BHGZ: Baihu Jia Guizhi decoction
CFA: Complete Freund's Adjuvant
HPLC: High-performance liquid chromatography
IL-1*β*: Interleukin-1 beta
RA: Rheumatoid arthritis
SUCNR1: Succinate receptor 1
SDH: Succinate dehydrogenase
TCM: Traditional Chinese Medicine.

## Supplementary Materials

Supplementary Materials Supplementary Figure 1: typical chromatogram of succinate, pyruvate, and fumarate in synovial tissue. A, mixed standard working solution including succinate (180 *μ*M), pyruvate (200 *μ*M), and fumarate (100 *μ*M). B, HPLC profile of synovial tissue.
