# Supplementary material for: Baihu Jia Guizhi Decoction Improves Rheumatoid Arthritis Inflammation by Regulating Succinate/SUCNR1 Metabolic Signaling Pathway
Source: Evid Based Complement Alternat Med. 2019 Dec 26;2019:3258572. doi: 10.1155/2019/3258572 (PMC6948314; doi:10.1155/2019/3258572)
Supplement: Supplementary Materials — Supplementary Figure 1: typical chromatogram of succinate, pyruvate, and fumarate in synovial tissue. A, mixed standard working solution including succinate (180 μM), pyruvate (200 μM), and fumarate (100 μM). B, HPLC profile of synovial tissue. [file 3258572.f1.docx]

Supplementary Figure 1: Typical chromatogram of succinate, pyruvate, and fumarate in synovial tissue. A, mixed standard working solution including succinate (180μM), pyruvate (200μM), and fumarate (100μM). B, HPLC profile of synovial tissue.
